# A Voxel-Based Morphometric MRI Study in Young Adults with Borderline Personality Disorder

**DOI:** 10.1371/journal.pone.0147938

**Published:** 2016-01-25

**Authors:** Xinhu Jin, Mingtian Zhong, Shuqiao Yao, Xiyu Cao, Changlian Tan, Jun Gan, Xiongzhao Zhu, Jinyao Yi

**Affiliations:** 1 Medical Psychological Institute, Second Xiangya Hospital, Central South University, 139 Renmin Middle Road, Changsha, Hunan, 410011, P.R. China; 2 Center for Studies of Psychological Application, School of Psychology, South China Normal University, 55 Zhongshan Road, Guangzhou, Guangdong, 510631, P.R. China; 3 Key Laboratory of Psychiatry and Mental Health of Hunan Province, Central South University, 139 Renmin Middle Road, Changsha, Hunan, 410011, P.R. China; 4 Department of Radiology, Second Xiangya Hospital, Central South University, 139 Renmin Middle Road, Changsha, Hunan, 410011, P. R. China; 5 National Technology Institute of Psychiatry, Central South University, 139 Renmin Middle Road, Changsha, Hunan, 410011, P.R. China; Central Institute of Mental Health, GERMANY

## Abstract

**Background:**

Increasing evidence has documented subtle changes in brain morphology and function in patients with borderline personality disorder (BPD). However, results of magnetic resonance imaging volumetry in patients with BPD are inconsistent. In addition, few researchers using voxel-based morphometry (VBM) have focused on attachment and childhood trauma in BPD. This preliminary study was performed to investigate structural brain changes and their relationships to attachment and childhood trauma in a homogenous sample of young adults with BPD.

**Method:**

We examined 34 young adults with BPD and 34 healthy controls (HCs) to assess regionally specific differences in gray matter volume (GMV) and gray matter concentration (GMC). Multiple regressions between brain volumes measured by VBM and attachment style questionnaire (ASQ) and childhood trauma questionnaire (CTQ) scores were performed.

**Results:**

Compared with HCs, subjects with BPD showed significant bilateral increases in GMV in the middle cingulate cortex (MCC)/posterior cingulate cortex (PCC)/precuneus. GMC did not differ significantly between groups. In multiple regression models, ASQ insecure attachment scores were correlated negatively with GMV in the precuneus/MCC and middle occipital gyrus in HCs, HCs with more severe insecure attachment showed smaller volumes in precuneus/MCC and middle occipital gyrus, whereas no negative correlations between insecure attachment and GMV in any region were found in BPD group. In addition, CTQ total scores were not correlated with GMV in any region in the two groups respectively.

**Conclusions:**

Our findings fit with those of previous reports of larger precuneus GMV in patients with BPD, and suggest that GMV in the precuneus/MCC and middle occipital gyrus is associated inversely with insecure attachment style in HCs. Our finding of increased GMV in the MCC and PCC in patients with BPD compared with HCs has not been reported in previous VBM studies.

## Introduction

Borderline personality disorder (BPD) is a highly prevalent axis II psychiatric disorder in general and clinical populations [1], typified by features such as pervasive instability in the regulation of emotion, self-image, interpersonal relationships and impulse control [2]. The estimated prevalence of BPD is 2% in the general population [3], 10% among psychiatric outpatients and 15%–25% among psychiatric inpatients [4].

BPD is also a paradigmatic disorder of adult attachment, with high rates of antecedent childhood maltreatment [5]. Several developmental models have suggested that BPD pathology (i.e., BPD or its features) is shaped by a combination of biological and environmental mechanisms, the latter of which include social and attachment-related disturbances [6]. Previous studies supported that insecure attachment styles are associated with personality disorders, wherein cluster B personality disorders (i.e., antisocial, narcissistic, especially borderline) are prominent [7,8]. Agrawal et al. [9] noted several reports of a significant, strong association between insecure attachment and BPD, notwithstanding variation among studies in measures used and attachment types examined. However, very few studies of BPD to date have examined neural patterns in relation to attachment, a basic behavioral system that processes relationship-based emotional experience and regulation in subjects with BPD [10].

Childhood trauma is another psychological characteristic that has been hypothesized to lead to BPD [11,12], which was found to associate with many adult psychiatric disorders, including affective, dissociative disorders, substance use disorders and sexual dysfunction [13]. Zanarini et al. [14] suggested that childhood maltreatment (i.e., emotional neglect, physical and/or sexual abuse) by a caregiver is among the most important psychosocial risk and prognostic factors for BPD pathology. Childhood exposure to physical or sexual abuse or severe neglect is related to anxious and avoidant adult attachment [15]. Adult disorganized or unresolved attachment has been related to maltreatment and physical abuse or neglect in childhood [16]. However, the neural correlate of attachment disturbance and childhood maltreatment in subjects with BPD is presently unknown. Therefore, using neuroimaging techniques to examine these two psychological characteristics is necessary to gain a comprehensive understanding of BPD.

Several neuroimaging techniques, such as positron emission tomography (PET) and region-of-interest (ROI) morphometry, have been used to enhance understanding of the psychobiology of BPD [17,18]. Most studies of brain volume in BPD have used a priori ROI approach, which enables precise detection of small volume differences [18,19]. While few studies to date have used voxel-based morphometry (VBM) [20], an unbiased, fully automatic technique believed to be superior to other approaches such as ROI analysis [21]. Assessing the whole-brain structural imaging data using Statistical Parametric Mapping (SPM), VBM does not require a priori definitions of anatomical areas, and is independent of hypotheses. Also, it is free of rater bias, inter-rater variability and highly efficient for large samples [22]. Given the advantages of VBM, several researchers have begun to focus on this modality for the examination of BPD [23–25].

The VBM studies of BPD conducted to date have revealed gray matter abnormalities in the frontal, temporal, parietal and limbic brain regions [26–28]. In the first study to employ VBM for this purpose, Rüsch et al. [25] found reduced gray matter volume (GMV) in the left amygdala in female patients with BPD compared with healthy controls (HCs). In a larger sample (60 female patients with BPD and 60 female HCs), Niedtfeld et al. [26] observed local differences in GMV in the amygdala, hippocampus, and fusiform and cingulate gyri. As volumetric abnormalities in the hippocampus are of major interest in the examination of BPD, many researchers have focused on this region. For instance, O’Neill et al. [29] found volume reductions in the right dorsolateral prefrontal cortex (DLPFC), right caudate and right hippocampus in patients with BPD compared with healthy subjects. Kuhlmann et al. [12] also found reduced GMV in the hippocampus and increased GMV in the hypothalamus in female patients with BPD compared with healthy participants, but no significant alteration in the amygdala or anterior cingulate cortex (ACC). To determine whether brain volume alterations exist in adolescents, Brunner et al. [30] compared adolescent patients with BPD with patients with other psychiatric disorders and HCs. They found reduced GMV in the DLPFC and orbitofrontal cortex in patients with BPD compared with HCs, but no significant GMV difference between the two patient groups. Völlm et al. [28] found GMV differences in the orbitofrontal cortex; middle frontal, precentral and postcentral gyri; temporal pole; and inferior and superior parietal cortices between male patients with BPD and male HCs. In a sample including males and females, Soloff et al. [22] observed significant bilateral reductions in gray matter concentration (GMC) in the ventral cingulate gyrus and several regions of the medial temporal lobe, including the hippocampus, amygdala, parahippocampal gyrus and uncus, in BPD patients compared with HCs (*n* = 34 each). In a further study, Soloff et al. [31] compared suicide attempters and non-attempters with BPD, as well as high- and low-lethality attempters, with HCs to identify neural circuits associated with suicidal behavior in BPD. They found significant differences in GMC in the insula, orbitofrontal gyrus and middle superior temporal cortex associated with suicidal behavior in male and female patients with BPD.

Although all of these above VBM studies have documented evidence of gray matter abnormality in patients with BPD, some results of VBM studies of BPD were inconsistent and even contradictory. For example, Labudda et al. [24] observed no volume difference in the whole brain between BPD patients and HCs in a recent VBM study. Also, increased and decreased GMC in the amygdala have been reported in adult patients with BPD [22,27]. In our opinion, possible reasons for such differences in previous research might include sample size (some studies included fewer than 10 patients with BPD [28]), sample heterogeneity (some samples have included patients with posttraumatic stress disorder (PTSD) or bipolar disorder (BD) as well as those with BPD [26,32]), and use of different statistical significance levels (*P* = 0.001 uncorrected in several studies [25,32]; *P* < 0.05 for the false discovery rate (FDR) or family wise error (FWE) for multiple comparisons [12,26,28,30]).

Previous VBM studies have rarely examined correlations between brain abnormalities and some important psychological characteristics of BPD, such as attachment and childhood trauma, especially the former. However, several neruoimaging studies have investigated the relationships between brain abnormalities and some psychological measures, and got meaningful results [33–37]. With structural imaging techniques, Tebartz van Elst L et al. [33] found reduced hippocampus and amygdala gray matter volumes in patients with BPD reporting traumatic attachment histories. With functional imaging studies, researchers investigated social attachment and demonstrated that pictures of loved ones would evoked cortical and subcortical responses, including those in the cingulate cortex, insula, basal ganglia and orbitofrontal cortex, in healthy subjects [34,35]. Skodol et al. [36] also found that BPD involves developmental or acquired brain dysfunction associated with early childhood traumatic experience. Meanwhile, associations between childhood maltreatment and brain gray matter volume reductions in the hippocampus [19], as well as in the insula and mesial frontal brain areas [37], were recently reported in large normal population samples. Therefore, examination of relationships between brain volume and attachment, as well as childhood trauma, in patients with BPD using VBM, an efficient exploratory technique for the study of brain–behavior relationships, is thus essential.

Overall, further studies with large, homogenous samples and more prudent analytical methods are necessary to clarify the differences in gray matter between patients with BPD and HCs. Thus, in this study, we used Diffeomorphic Anatomical Registration Through Exponentiated Lie (DARTEL) Algebra [38], an improved VBM method that can achieve inter-subject brain image registration more accurately, to assess regionally specific differences in GMV and GMC between patients with BPD and control subjects. We also investigated relationships between gray matter volumes and measures of attachment and childhood trauma in multiple regression models. To our knowledge, few BPD studies to date have used DARTEL Algebra. Based on the results of published studies, we hypothesized that VBM analyses would demonstrate gray matter abnormalities in the prefrontal, temporal and limbic areas in subjects with BPD compared with HCs. We also supposed that insecure attachment correlated with gray matter volumes in the prefrontal gyrus and cingulate cortex, whereas the childhood trauma correlated with gray matter volumes in limbic areas.

## Methods

### Participants

A total of 34 right-handed young adults with BPD were recruited from outpatient clinics affiliated with the Second Xiangya Hospital of Central South University, Changsha, Hunan, China. Thirty-four right-handed volunteers were recruited for the HC group. The Ethics Committee of the Second Xiangya Hospital of Central South University approved the study. All subjects were made aware of the purpose of the study and provided written informed consent.

Two well-trained psychiatrists made diagnoses of BPD independently based on the structured clinical interview for axis II disorders (SCID-II) of the Diagnostic and Statistical Manual of Mental Disorders, Fourth Edition (DSM-IV) [39]. The psychiatrists rated each symptom item as absent (0), subclinical (1) or clinically present (2) based on the SCID-II users’ guide. Every patient also received the Structured Clinical Interview for DSM-IV Axis I disorders (SCID-I) by two psychiatrists to exclude Axis I disorders [40]. Exclusion criteria were: past or current axis I diagnosis of schizophrenia, delusional (paranoid) disorder, schizoaffective disorder, BD or psychotic depression; physical disorder of known psychiatric consequence (e.g., hypothyroidism, seizure disorder, brain injury); and borderline mental retardation. Participants’ medical records were reviewed when available to confirm fulfilment of the inclusion or exclusion criteria. None of the patients had received psychiatric treatment.

HCs were recruited by advertisements from the surrounding community. To rule out any DSM-IV axis I or axis II disorder, two well-trained psychiatrists also interviewed HCs with SCID-I and SCID-II [39,40]. Control subjects were physically healthy individuals with no past or current history of any DSM-IV axis I or axis II disorder, no current medical problem, and no history of psychiatric disorders among first-degree relatives.

Depression and anxiety severity were rated using the Chinese version of the Center for Epidemiologic Studies Depression Scale [41] and the State-Trait Anxiety Inventory [42], respectively. The Attachment Style Questionnaire (ASQ) [43], which yields scores for five scales or dimensions, was used to assess attachment style. The confidence scale of the ASQ reflects secure attachment, and the other four scales (discomfort, approval, preoccupied and secondary) represent particular aspects of insecure attachment. In this study, only insecure attachment score was used, which ranges from 32 to 192. The Childhood Trauma Questionnaire (CTQ) [44] was used to assess childhood trauma, including sexual abuse, emotional abuse, emotional neglect, physical abuse and physical neglect. While in this study, only CTQ total score was used, which ranges from 28 to 140. All the psychological measures used in this study have shown good reliability and validity [43–45].

### Magnetic resonance imaging acquisition

Magnetic resonance imaging (MRI) was performed using a Philips Ingenia scanner operating at 3.0 T. All participants were asked to remain quiet during scanning. Ear plugs and foam pads were used to minimize noise and head motion. MRI data were acquired using a single T1-weighted turbo-field echo sequence with the following acquisition parameters: repetition time = 6.7 ms, echo time = 3.1 ms, flip angle = 8°, field of view = 240 mm, slice thickness = 1.0 mm, acquisition matrix = 240 × 240 and voxel size = 1.0 × 1.0 × 1.0 mm^3^.

### MRI data analysis

MR images were cropped and reoriented following the anterior–posterior commissure line with MRIcro (http://www.cabiatl.com/mricro/mricro/mricro.html). The resulting images (including brain, cerebellum and brainstem) were then processed using SPM 8 software (www.fil.ion.ucl.ac.uk/spm). For each image, the anterior commissure was manually set as the origin of the spatial coordinates using SPM 8.

Images were registered to a group-specific common space following the DARTEL Algebra procedure in SPM 8 [35]. The procedure involves the following steps: (1) segmentation of gray matter tissue maps for each subject using the standard unified segmentation model of SPM 8; (2) alignment of gray matter tissue maps to a standard space through rigid-body transformations; (3) generation of gray matter DARTEL templates through a six-step iterative procedure, which involves the creation and refining of the templates and associated image warping fields (briefly, a first template is created by normalizing initial images to a standard template and averaging; subsequent templates are then defined iteratively by normalizing images to the template obtained at the previous iteration, and the final deformation is then parameterized as a warping field); (4) non-linear warping of the individual gray matter images to the corresponding DARTEL template using the warping fields computed in the previous step; (5) modulation of warped gray matter images to preserve the original GMVs and GMCs; and (6) smoothing of the modulated images with a Gaussian kernel of 8 × 8 × 8 full width at half-maximum to improve the normality of data, thereby increasing the power of subsequent statistical analyses. As the templates generated by DARTEL Algebra represent the average shape of the subjects included in the analysis and the reference space thus differs generally from Montreal Neurological Institute (MNI) space, normalized and modulated gray matter images were converted to MNI space prior to smoothing.

### Statistical analysis

Demographic and clinical characteristics of participants in the two groups were compared using chi-squared tests for categorical variables and independent-sample *t*-tests for continuous variables. Statistical analyses were performed using the Statistical Package for Social Sciences, version 17.0 (SPSS Inc., Chicago, IL, USA).

Volumetric data from the BPD and HC groups were compared using *t*-tests. Images were thresholded at an absolute level of 0.1, resulting in the inclusion only of voxels exceeding the threshold. Whole-brain analyses were generated separately for gray matter and corrected for multiple comparisons. A FDR threshold was determined from the observed *P*-value distribution and hence adapted to the size of the effect in the data. An extent threshold for voxel clusters of at least *k* = 50 voxels and a significance threshold of *P* < 0.05 were chosen.

Correlations between gray matter volume and psychological assessment (ASQ, CTQ) scores were assessed using a multiple regression model performed with SPM 8, adjusted for subjects’ total intracranial volume (TIV). TIV was obtained by summing the volumes of gray matter, white matter and cerebrospinal fluid using the “get_totals” function (www.cs.ucl.ac.uk/staff/G.Ridgway/vbm/). In this study, we only used gray matter volume in a multiple regression model for correlation analyses [38]. Since insecure attachment and childhood trauma are probably highly related, in multiple regression models, we first conducted regression analyses for insecure attachment scores of ASQ with GMV in BPD group, adjusted for CTQ total socres and TIV. Then, we conducted regression analyses for CTQ total socres with GMV in BPD group, adjusted for insecure attachment scores of ASQ and TIV. And the same analysis procedures above were repeated in HC group.

## Results

### Demographic and clinical characteristics

The demographic and clinical characteristics of all subjects are summarized in Table 1. Participants in the BPD and HC groups were well matched, with no significant differences in age, gender, education, depression symptomatology, anxiety severity or TIV (Table 1). ASQ insecure attachment scores and CTQ total scores were significantly higher in the BPD group than in the HC group.

**Table 1:** Demographic and clinical characteristics of patients with borderline personality disorder and healthy controls.

	BPD (Mean±SD) N = 34	HC (Mean±SD) N = 34	*t* Value/ChiSq.	*p* Value
**Age(years)**	22.91±1.16	22.91±0.79	0.000	1.000
**Gender (male: female)**	17: 17	15: 19	0.235	0.628
**Education(years)**	16.32±0.73	16.65±0.73	-1.826	0.072
**CES-D**	34.29±8.25	30.82±8.72	1.685	0.097
**SAI**	35.18±11.14	30.85±8.40	1.809	0.075
**TAI**	39.79±9.28	36.38±8.19	1.607	0.113
**Insecure attachment** ^a^	115.62±16.97	95.57±11.72	5.668	0.000
**CTQ**	39.79±12.31	29.60±4.07	4.583	0.000
**TIV**	1448.69±141.91	1422.72±117.81	0.821	0.415

BPD = Borderline Personality Disorder; HC = Healthy Control; CES-D = Center for Epidemiologic Studies Depression Scale (total scores range from 20 to 80); SAI = State Anxiety Inventory (total scores range from 20 to 80); TAI = Trait Anxiety Inventory (total scores range from 20 to 80); CTQ = Childhood Trauma Questionnaire (total scores range from 28 to 140); TIV = Total Intracranial Volume.

^a^ four subscales (Discomfort, Approval, Preoccupied, Secondary) of Attachment Style Questionnaire are combined to make Insecure attachment (total scores range from 32 to 192)

### Group differences in gray matter abnormalities

**Fig 1:**
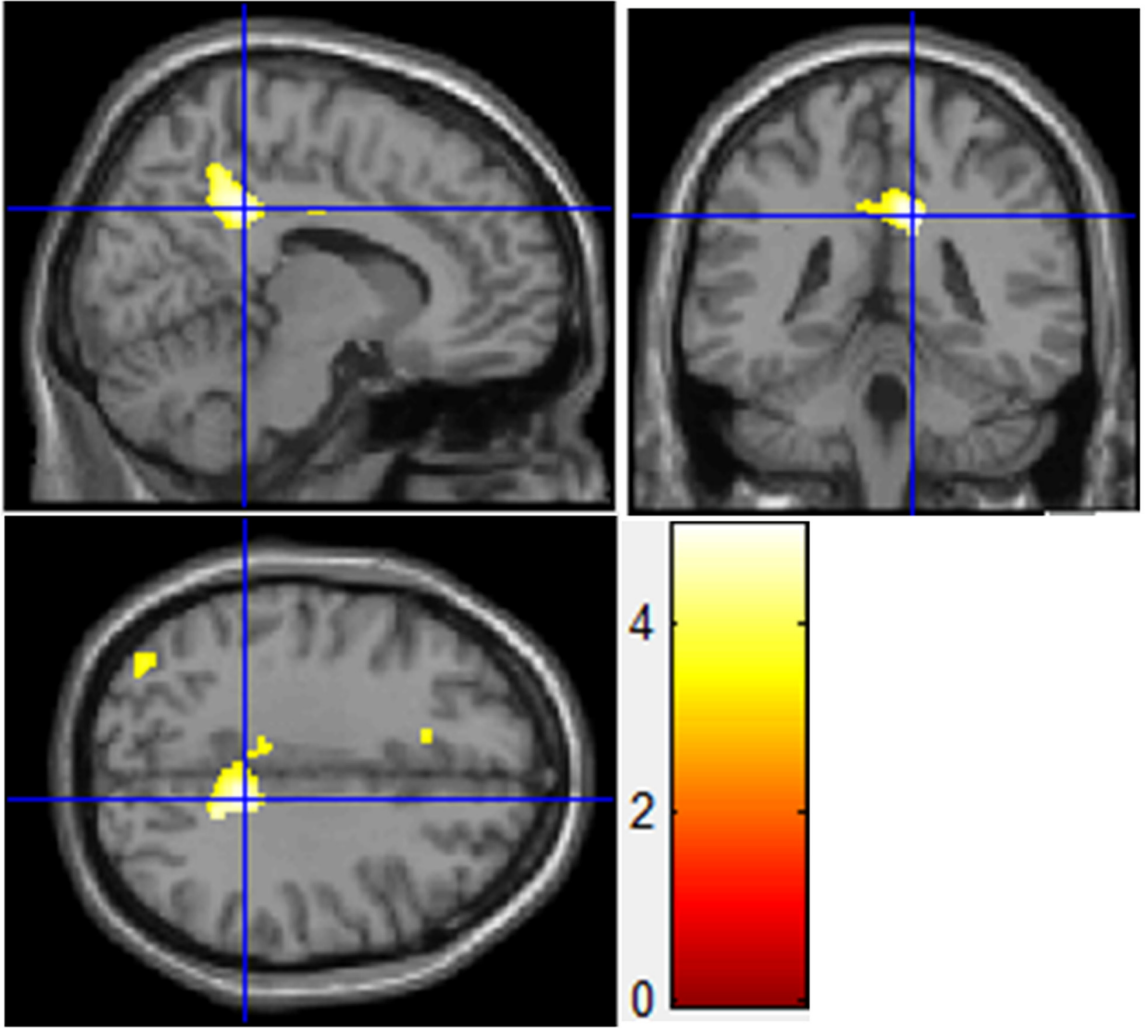
Gray matter volume differences of the right middle cingulate cortex/posterior cingulate cortex/precuneus. Gray matter volume enlargement was found in the right middle cingulate cortex/posterior cingulate cortex/precuneus of patients with borderline personality disorder compared with healthy controls (MNI coordinates, x = 11, y = –40, z = 34; P_FDR-corrected_ = 0.011; extent threshold, 1239 voxels).

Compared with HCs, subjects with BPD showed significant bilateral GMV increases in the middle cingulate cortex (MCC)/posterior cingulate cortex (PCC)/precuneus (Table 2, Fig 1), especially on the right side. GMC did not differ significantly between the BPD and HC groups. In the reverse contrast (HC > BPD), we found no significant difference in GMV or GMC between groups.

**Table 2:** Results of whole-brain voxel-based analyses of gray matter volume.

Test and contrast	Regions	K	P(FDR)	t	Z score	MNI [x y z]		
**Two-sample t-Test: BPD>HC**	MCC/PCC/ Precuneus	1239	0.011	5.07	4.64	11	-40	34
**Regression Analysis ASQ in HC group**	Precuneus/ MCC	1404	0.002	6.50	5.09	-17	-48	43
**(Negative correlation)**	Middle occipital gyrus	945	0.008	5.21	4.36	41	-81	-5

BPD = Borderline Personality Disorder; HC = Healthy Control. MCC = Middle cingulate cortex; PCC = Posterior cingulate cortex.

### Correlations between morphometric data and psychometric findings

Multiple regression models showed different involvement of GMV associated with insecure attachment and childhood trauma. Significant negative correlations were present between ASQ insecure attachment scores and GMV in the precuneus/middle cingualte cortex (MCC) (Table 2) and middle occipital gyrus (Table 2) in HC group, HCs with more severe insecure attachment showed smaller volumes in precuneus/MCC and middle occipital gyrus, whereas no negative correlations between insecure attachment and GMV in any region were found in BPD group. In addition, CTQ total scores were not correlated with GMV in any region either in the two groups.

## Discussion

The aim of this study was to investigate GMV and GMC abnormalities in the whole brain in a homogenous sample of young adult patients with BPD compared with HCs and relationships between gray matter volume and insecure attachment as well as childhood trauma, using VBM-DARTEL Algebra. VBM revealed that young adults with BPD displayed significantly increased GMV bilaterally in the MCC/PCC/precuneus compared with healthy subjects. GMV were correlated negatively with insecure attachment scores in the precuneus/MCC and middle occipital gyrus in HCs, HCs with more severe insecure attachment showed smaller volumes in precuneus/MCC and middle occipital gyrus.

### Increased MCC volume, an indicator of deficits in emotional-cognitive interplay in BPD

Many researchers have found the MCC (called the dorsal anterior cingulate cortex in many studies), suggested to be the “cognitive” part of the ACC in emotional–cognitive interplay [46], to be hyperactivated in patients with BPD [47]. As a proportion of ACC, MCC is one of the cognitive and monitoring regions involved in salience detection, attention regulation, and cognitive control [48]. Functions related to the anticipation of unpleasant stimuli [49], as well as the integration and control of emotional stimuli [50], have also been linked to the MCC.

In the present study, we observed increased GMV in the MCC in subjects with BPD compared with HCs. In a previous study, Faymonville et al. [51] found that the juncture of anterior midcingulate cortex (aMCC) and posterior midcingulate cortex (pMCC) had enhanced activation when the intensity and unpleasantness of noxious stimuli was increased. Vogt et al. [52] demonstrated that the activity in aMCC would increase as a result of the high level of fear activations in this region. Using functional MRI, Buchheim et al. [10] found that the activation of patients with BPD was located in the anterior midcingulate cortex (aMCC) during telling individual stories, which was consistent with a fluorodeoxyglucose-PET study demonstrating increased baseline ACC metabolism extending from the aMCC into the medial prefrontal cortex in patients with BPD compared with HCs [17]. And Buchheim et al. [10] interpreted their finding of clearly more dorsal aMCC activation as an indicator of unsuccessful coping with emotional pain and a neural signature of pain and fear in these patients. Thus, involvement of aMCC in pain and fear is feasible, which was consistent with the previous studies. Moreover, increased activation of the dorsal anterior cingulate cortex in BPD has already been reported during response inhibition [53]. Given that increased gray matter in BPD (compared with control subjects) is likely to be related in a straightforward manner to patterns of increased functional activation, as the blood oxygen level–dependent signal appears to be related most closely to local field potentials, which arise primarily from dendritic membrane potentials in a local region [54], we can reasonably make a hypothesis that increased GMV in MCC might relate to increased activation in MCC, and might be an indicator of deficits in emotional–cognitive interplay in young adult patients with BPD.

### Abnormal PCC/precuneus size, abnormality in the default mode circuit in BPD

As a central node of the so-called default mode circuit in the brain, the PCC (with the precuneus) has been identified as a major hub involved in non-task-based attention and readiness of response to external and internal environments [55]. The PCC functions as a “convergence node” within the default mode network (DMN), where information integration and the interaction between different subsystems are facilitated [56]. An abnormality in this region may signify an abnormality in the default mode circuit, which could disrupt mental processes, leading to distractability and decreased attention to internal mental processes, as seen frequently in mania [55]. The precuneus has been shown to be activated during imagination of one's own actions or movements and during tasks requiring introspection, self-evaluation and reflection upon one's own personality and mental state [57]. Moreover, it is involved in visuospatial imagery, episodic memory retrieval, self-processing and consciousness (behavioral correlates of the precuneus) [57]. Along with the PCC, the precuneus has been implicated in self-referential processing and first-person perspective [50,57], features consistent with the tendency for patients with BPD to become emotionally overinvolved in interpersonal situations.

In the present study, we found larger GMV in the PCC/precuneus in patients with BPD than in HCs. Although no previous VBM study has demonstrated volumetric abnormality in the PCC in BPD, brain activity in this region is more pronounced in patients with BPD than in controls when anticipating negative pictures [58]. Scherpiet et al. [58] suggested that heightened PCC activation during the anticipation of unspecific, non-self-related emotional stimuli fit well with the prominent self-reference in everyday life situations that characterizes BPD. This aspect may explain a stronger emotional engagement in patients with BPD compared with healthy participants [58]. PCC activation has also been found to be a correlate of anger processing [50] and the processing of threat-related words [59]. Thus, increased GMV in the PCC might play the same role as heightened PCC activation in patients with BPD. With regard to precuneus, in a previous VBM study of patients with BPD, Soloff et al. [22] found increased GMCs in a very large area of the right cerebrum extending from the right superior frontal gyrus posteriorly and across the parietal lobe to the precuneus, similar to our results. Moreover, almost all studies have shown the spreading of activation to the precuneus during mental imagery [57]. Stress-related dissociative symptoms are known to occur in about 75% of individuals with BPD [36], and depersonalization is a major dissociative symptom, according to the SCID for dissociative disorders [60]. Irle et al. [18] reported that a larger right precuneus was correlated positively with stronger depersonalization in subjects with BPD. Simeon et al. [61] reported that dissociative symptom severity in individuals with depersonalization disorder was correlated with increased metabolic activity of the precuneus. Dissociative states may be considered to be pathological conscious states, potentially involving precuneus recruitment. In addition, the PCC and precuneus are involved in conscious processing of information and self-reflection [62]. Broyd et al. [63] suggested that enhanced connectivity of the precuneus/PCC reflects a disturbance of self-referential and emotional processing of pain in BPD. In a previous study, Logothetis and Wandell [54] have demonstraded that gray matter increase might relate to patterns of increased functional activation in a straightforward manner. Therefore, our results of increased GMV in PCC/precuneus might suggest heightened PCC/precuneus activation in patients with BPD compared with HCs, which needs further studies to verify.

### Structural changes associated with insecure attachment style and childhood trauma

The results of multiple regression models in the present study showed different involvement of GMVs associated with insecure attachment style and childhood trauma, which requires further analysis. On the one hand, we found no correlations between CTQ total scores and GMV in any region in either of the two groups. Using VBM method, Labudda et al. [24] did not find a relationship between childhood maltreatment and the BPD patients’ brain volumes, which was consistent with our results in BPD group. However, using VBM-DARTEL, Dannlowski et al. [37] found the morphometric analysis yielded reduced gray matter volumes in the hippocampus, insula, orbitofrontal cortex, anterior cingulate gyrus, and caudate in HC subjects with high CTQ scores, which we did not find in our HC group.

On the other hand, we only found negative correlations between insecure attachment scores and GMV in precuneus/MCC and middle occipital gyrus in HC group, but not in BPD group, which suggests that HCs with more severe insecure attachment showed smaller volumes in precuneus/MCC and middle occipital gyrus. In fact, some neuroimaging studies have investigated neural correlates of adult insecure attachment by means of affective and/or attachment-related stimuli, and demonstrated the relationships between dorsal anterior cingulate cortex (MCC) and insecure attachment [64,65]. In a recent research, Schneider-Hassloff et al. [66] measured brain activation using fMRI in healthy subjects to test how the adult insecure attachment styles modulate neural correlates of mentalizing. They found a strong activation of the mentalizing network, including bilateral precuneus, (anterior, middle, and posterior) cingulate cortices. They also found that insecure attachment styles correlated with task-associated neural activity in the mid-cingulate cortex. Our results in HCs provide further evidences for the neural correlate of insecure attachment in healthy subjects. Based on the results in our multiple regression analyses and other relevant research results above, we could make a suppostion that the MCC might play a central role in insecure attachment style in HCs. However, we did not find significant negative correlations between insecure attachment and GMV in precuneus/MCC in BPD group, which was not consistent with our hypothesis and might be due to the GMV increases in BPD group. Negative correlations between insecure attachment style and GMV in precuneus/MCC were found in HCs, which suggested HCs with more severe insecure attachment showed smaller volumes in precuneus/MCC, while the enlarged GMVs in the MCC/precunes in young adults with BPD might weaken the negative correlations between insecure attachment and GMV.

The results of our study are subject to some limitations. First, the analysis was based on a massive voxel-by-voxel univariate analysis, which exhibits lower accuracy during segmentation of some subcortical structures, such as the thalamus. Second, we described findings in terms of mean differences in indices (GMV and GMC) between the BPD and HC groups. These mean differences can be important when describing and identifying regions that play a role in BPD, but they do not provide information about individual cases or clinical applications in individual subjects. Third, because the young patients with BPD in our sample had no comorbid axis I/II disorders and may be not representative of other BPD populations [4], the generalizability of results in our study remains to be determined.

## Conclusions

In summary, this study is the first to report GMV enlargement in the MCC and PCC in patients with BPD using VBM. Our preliminary finding of larger precuneus in BPD is consistent with the results of previous MRI and PET studies. Further studies are needed to replicate these findings and to verify the relationships between measures of attachment style and childhood trauma and volumetric abnormalities in patients with BPD.

## Supporting Information

S1 Dataset Data of demographic and clinical characteristics of all subjects and data of structural MRI. (XLS)
